# Stereotactic radiosurgery in symptomatic circumscribed choroidal hemangiomas

**DOI:** 10.1007/s00417-026-07231-2

**Published:** 2026-04-27

**Authors:** Alper Kahvecioglu, Irem Koc, Mustafa Cengiz, Ayca Yilmaz, Ekim Gumeler, Hayyam Kiratli, Gozde Yazici

**Affiliations:** 1 Department of Radiation Oncology, Hacettepe University Faculty of Medicine, Ankara, Turkey; 2 Department of Ophthalmology, Hacettepe University Faculty of Medicine, Ankara, Turkey; 3 Department of Radiology, Hacettepe University Faculty of Medicine, Ankara, Turkey

**Keywords:** Benign diseases, Choroidal hemangioma, Stereotactic radiosurgery, Radiotherapy, Visual acuity

## Abstract

**Purpose:**

To evaluate visual outcomes following stereotactic radiosurgery (SRS) in patients with symptomatic circumscribed choroidal hemangioma (cCH).

**Methods:**

This retrospective study included 30 patients with symptomatic cCH treated with SRS between 2014 and 2025. SRS was performed using either the CyberKnife or ZAP-X platform depending on the treatment period. Visual outcomes were assessed by changes in best-corrected visual acuity (BCVA). A lower score on the logarithm of the minimum angle of resolution (logMAR) scale indicates better vision.

**Results:**

The median patient age was 46 years. Ten patients (33.3%) were treatment-naive, while 20 (66.7%) had prior therapies. A single-fraction dose of 10 Gy was delivered in 29 patients (96.7%), and one patient received 14 Gy. Median BCVA improved from 1.9 logMAR (range, 0.1–3.0) at baseline to 1.3 logMAR (range, 0.1–3.0) after SRS (*p* = 0.01). Among 26 patients with baseline subretinal fluid, 12 (46.1%) showed complete and 10 (38.5%) partial resolution. Of six patients with retinal detachment (RD), three (50.0%) achieved anatomical resolution, but only one showed visual improvement. Logistic regression suggested a trend toward reduced likelihood of visual improvement after SRS in patients with baseline RD (OR: 0.121, *p* = 0.063). No clinically significant acute toxicity was observed. Two patients (6.7%) experienced grade 3 late visual acuity decrease, associated with foveal atrophy despite favorable anatomical response.

**Conclusions:**

SRS appears to be a safe and effective treatment for symptomatic cCH, providing visual improvement or stabilization in most cases. However, baseline RD may predict poorer visual outcomes, even with anatomical recovery. Early diagnosis and timely treatment may help optimize visual results and guide patient selection.

## Key messages

*****What is known***:**

- Circumscribed choroidal hemangioma (cCH) is a benign vascular tumor that can cause significant visual loss when associated with subretinal fluid or retinal detachment.
- Radiotherapy, including plaque brachytherapy and external beam techniques, has been used for refractory or recurrent cases with variable visual outcomes.

*****What is new***:**

- This study represents the largest series to date evaluating non-invasive stereotactic radiosurgery (SRS) for symptomatic cCH.
- SRS achieved significant improvement in best-corrected visual acuity (BCVA) and high rates of subretinal fluid resolution, demonstrating its effectiveness.
- The presence of baseline retinal detachment was associated with a lower likelihood of visual improvement, suggesting the importance of early diagnosis and timely intervention before irreversible retinal damage occurs.

## Introduction

Circumscribed choroidal hemangioma (cCH) is a benign and generally sporadic vascular tumor of the choroid that may cause progressive visual loss, particularly when complicated by subretinal fluid (SRF), retinal detachment (RD), or macular involvement [1]. While asymptomatic cases can often be managed conservatively with observation, symptomatic lesions usually require treatment to preserve or improve visual function. A range of therapeutic modalities has been explored for symptomatic cCH, including photodynamic therapy (PDT), laser photocoagulation, transpupillary thermotherapy (TTT), and radiotherapy (RT) [2–6]. RT may be delivered via plaque brachytherapy or external beam radiotherapy (EBRT). In addition to its role in refractory cases, EBRT, including fractionated photon therapy, proton beam therapy, and stereotactic techniques, may also be considered as a first-line option in selected patients with large or symptomatic lesions [7–11].

In recent years, stereotactic radiosurgery (SRS) has emerged as a precise, non-invasive EBRT technique, delivering high-dose, conformal radiation in one or a few fractions using platforms such as Gamma Knife, CyberKnife or ZAP-X [12–16]. Although SRS has shown promising results in the treatment of various intraocular tumors, including uveal melanomas and choroidal metastases, data regarding its use in cCH remain scarce and mostly limited to case reports or small single center series [17–19]. Moreover, the relationship between anatomical resolution and visual outcomes following SRS in cCH is not well established, and clinical predictors of visual response remain unknown. In this study, we sought to assess the effectiveness of SRS in improving visual and anatomical outcomes in symptomatic cCH, and to investigate clinical parameters that may predict visual response following treatment.

## Methods

### Study design and patient population

A retrospective evaluation was conducted on 43 patients diagnosed with cCH who underwent SRS at our institution between 2014 and 2025. Inclusion criteria were: (1) a confirmed diagnosis of cCH based on ophthalmologic evaluation and multimodal imaging; (2) presence of visual symptoms attributable to the lesion; and (3) availability of best-corrected visual acuity (BCVA) measurements both before and after SRS. Patients previously treated with PDT and/or TTT were eligible if SRS was later administered for persistent or recurrent disease. Patients were excluded if they had the diffuse subtype of the tumor, inadequate follow-up (< 3 months), or incomplete clinical documentation (*n* = 13). After exclusions, 30 patients with cCH were deemed eligible and included in the final analysis.

### Stereotactic radiosurgery

All patients were treated in the supine position using a thermoplastic mask for head immobilization. To minimize ocular movement, a peribulbar injection of lidocaine was administered approximately 30 min before both simulation and treatment. High-resolution planning involved 1 mm slice thickness images obtained from both simulation computed tomography (CT) and magnetic resonance imaging (MRI). Gross tumor volume (GTV) was defined as the contrast-enhancing lesion on planning scans. The median GTV was 240 mm3 (range: 81–365 mm3). The median prescribed dose was 10 Gy (range: 10–14 Gy), which represents our institutional standard based on published SRS series reporting favorable safety and efficacy [4, 12]. All treatments were delivered in a single fraction and targeted directly to the GTV without additional margins, using either CyberKnife or ZAP-X platforms.

### Visual and anatomical outcomes

BCVA was measured using the standard Snellen chart and converted into logarithm of the minimum angle of resolution (logMAR) values for statistical analysis. Lower logMAR values represent superior visual acuity. For patients with severely impaired vision, counting fingers, hand motion, light perception, and no light perception were assigned logMAR values of 1.9, 2.3, 2.7, and 3.0, respectively, based on previously established conventions [20–22]. BCVA was recorded both prior to treatment and at the last follow-up visit. The presence and resolution of SRF and RD were documented, along with an assessment of tumor size. SRF was defined as posterior pole fluid detectable only on optical coherence tomography (OCT), whereas RD was classified when an overt detachment was evident on fundoscopic examination. Tumor response was assessed according to changes in tumor thickness and basal dimensions on follow-up imaging. Tumor height and basal diameter were assessed using ultrasound (US), which served as the primary modality for evaluating tumor evolution. A partial response was defined as a measurable reduction in either parameter, while complete regression was recorded when both tumor thickness and basal dimensions returned to normal or became no longer detectable. Stable disease referred to unchanged measurements, and progression was defined as any increase in size.

### Statistical analysis

All statistical analyses were performed using IBM SPSS Statistics version 23.0 (IBM Corp., Armonk, NY, USA). Descriptive statistics, including frequencies, percentages, medians, and ranges, were used to summarize demographic and clinical characteristics. The normality of continuous variables was assessed using the Shapiro–Wilk test. For comparison of pre- and post-treatment BCVA, Wilcoxon signed-rank test was applied. Follow-up was calculated from the initiation date of SRS to the last follow-up. Visual improvement was defined as a reduction in logMAR value of ≥ 0.2 at the last follow-up. To identify potential predictors of visual improvement, univariate logistic regression analyses were initially performed for relevant clinical variables. Variables with a p-value < 0.10 were intended to be included in a subsequent multivariate logistic regression model to evaluate their independent association with visual improvement. However, since only one variable met this threshold in the univariate analysis, multivariate modeling was not conducted. A two-tailed p-value < 0.05 was considered statistically significant for all tests.

## Results

### Patient, tumor and treatment characteristics

Table 1 summarizes the baseline characteristics of the study cohort, which comprised 30 patients (18 males [60.0%] and 12 females [40.0%]) with a median age of 46 years (range: 18–66 years). SRS was administered as the primary treatment in 10 patients (33.3%), while 20 patients (66.7%) had previously undergone PDT and/or TTT. Most patients (*n* = 29, 96.7%) received a single-fraction dose of 10 Gy. A higher dose of 14 Gy was administered in one patient (3.3%) based on physician discretion. Baseline imaging revealed SRF in 26 patients (86.7%) and RD in 6 patients (20.0%). In the 13.3% (*n* = 4) without baseline SRF, all had previously received PDT or TTT and were referred for SRS due to persistent visual symptoms and lack of treatment response, despite lack of exudative signs.

**Table 1:** Patient, tumor and treatment characteristics

Characteristics	Number (%)
Age, median (range)	46 years (18–66 years)
Gender Male Female	18 (60.0) 12 (40.0)
Prior treatment before SRS Yes No	20 (66.7) 10 (33.3)
SRF before SRS Yes No	26 (86.7) 4 (13.3)
RD before SRS Yes No Unknown	6 (20.0) 23 (76.7) 1 (3.3)
BCVA before SRS, median (range)	1.9 logMAR (0.1-3 logMAR)
GTV, median (range)	240 mm^3^ (81–365 mm^3^)
SRS dose 10 Gy in 1 fraction 14 Gy in 1 fraction	29 (96.7) 1 (3.3)

Abbreviations: *BCVA* best-corrected visual acuity, *GTV *gross tumor volume, *RD *retinal detachment, *SRF* subretinal fluid, *SRS* stereotactic radiosurgery

Prior to treatment, the majority of patients presented with advanced visual impairment. Baseline BCVA was recorded as counting fingers in 8 patients (26.7%), hand motion in 7 (23.3%), light perception in 1 (3.3%), and no light perception in 2 (6.7%). The median baseline BCVA was 1.9 logMAR (range: 0.1–3.0), and only 8 patients (26.6%) had a BCVA better than 1.0 logMAR.

### Visual outcomes following SRS

**Fig. 1:**
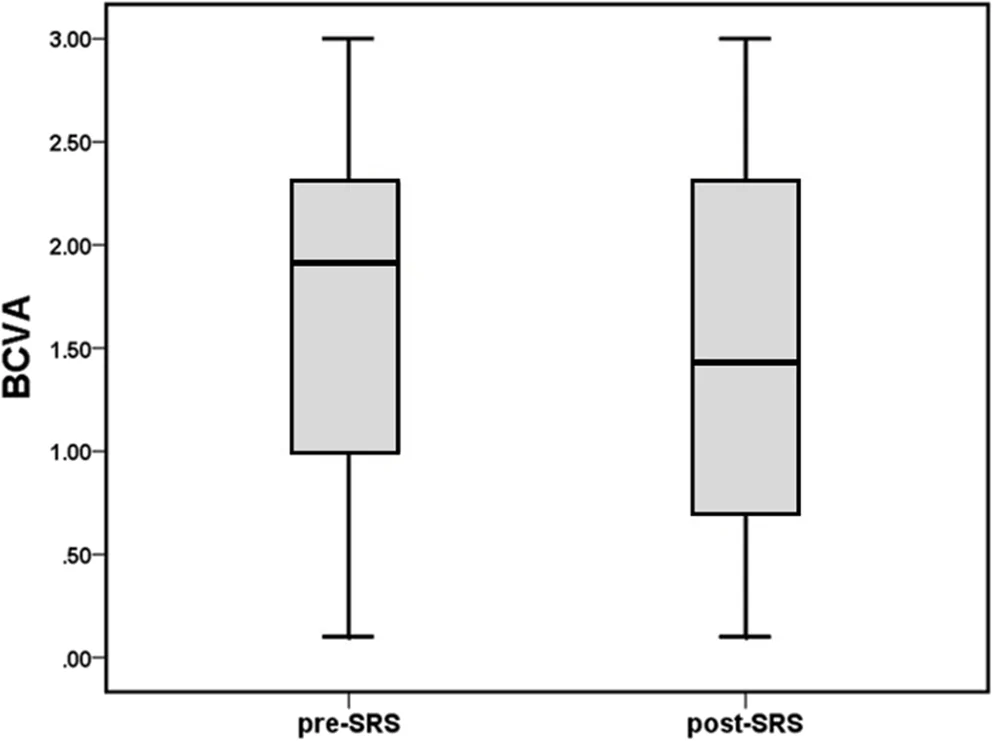
Boxplot showing logMAR best-corrected visual acuity before and after stereotactic radiosurgery. *Lower logMAR values represent superior visual acuity

The median follow-up duration was 20.8 months (range: 3.15–96.6 months), with 25 patients (83.3%) followed for more than a year. At last follow-up, BCVA improved by at least 0.2 logMAR in 15 patients (50.0%), remained stable in 13 (43.3%), and worsened in 2 (6.7%). Overall, the median BCVA improved, decreasing from 1.9 to 1.3 logMAR after SRS (Fig. 1, *p* = 0.01). Among patients who demonstrated a visual improvement, median BCVA decreased from 1.9 logMAR (range: 0.3–2.7) to 0.7 logMAR (range: 0.1–2.3).

### Anatomical outcomes following SRS

**Fig. 2:**
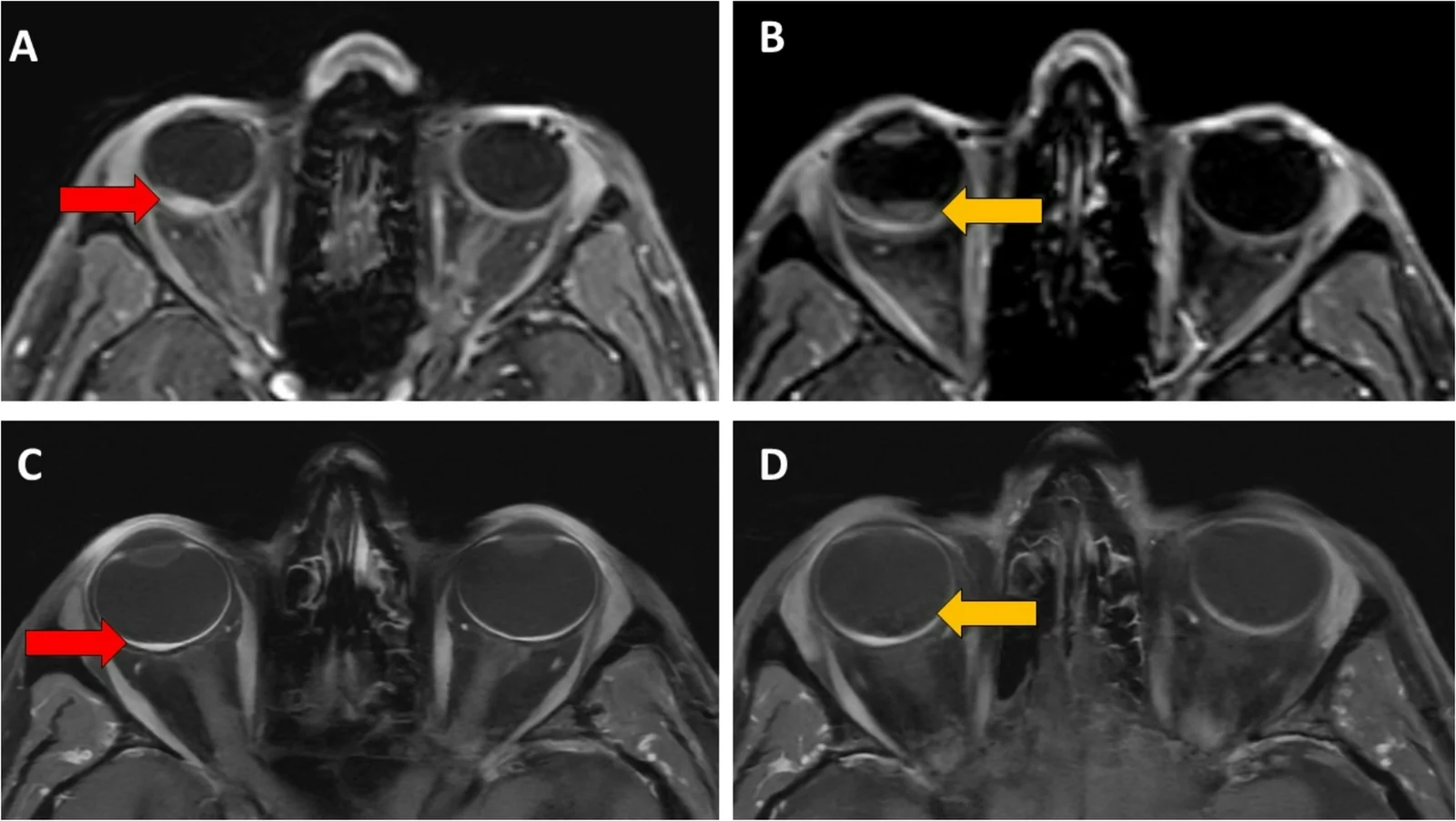
Representative orbital images of a patient before and after SRS (**A**) Pre-SRS axial image showing an enhancing choroidal lesion (red arrow). (**B**) Subretinal fluid is visible prior to treatment (orange arrow). (**C**) Post-treatment image demonstrating near-complete regression of the lesion. (**D**) Complete resolution of subretinal fluid following SRS (orange arrow)

Among 26 patients with baseline subretinal fluid (SRF), resolution was observed in 22 (84.6%), including complete resolution in 12 (46.1%) and partial resolution in 10 (38.5%). Among the 6 patients with baseline RD, 3 (50.0%) achieved complete anatomical resolution. Anatomical tumor response following SRS included complete response in 2 patients (6.7%), partial regression in 12 (40.0%), and stable disease in 14 (46.6%). In 2 patients (6.7%), tumor response could not be assessed. Representative pre- and post-treatment orbital MRI images of a patient demonstrating partial tumor regression and complete resolution of SRF are presented in Fig. 2.

### Predictors of visual improvement following SRS

**Fig. 3:**
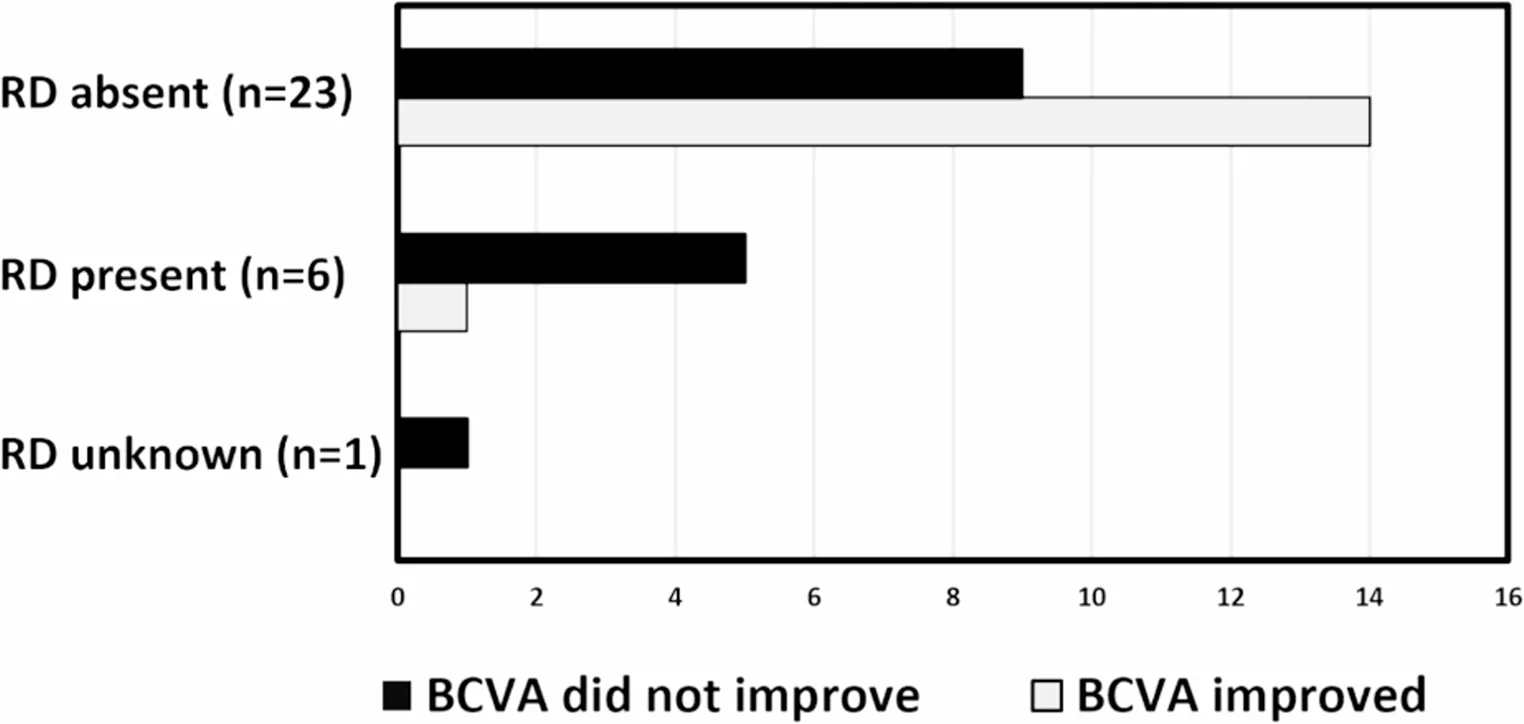
Number of patients with and without visual improvement, stratified by baseline retinal detachment (RD) status

Univariate logistic regression analyses were performed to identify potential predictors of visual improvement (Table 2). Among the variables analyzed, the presence of baseline RD showed a non-significant trend toward a negative association with visual improvement (OR = 0.121, 95% CI = 0.013–1.120, *p* = 0.06). No other variables, including age, sex, tumor volume, prior treatments, baseline SRF or baseline BCVA, were found to be associated with visual improvement following SRS. Among the six patients who presented with RD at baseline, anatomical resolution was achieved in three; however, only one of these patients experienced visual improvement following SRS (Fig. 3).

**Table 2:** Univariate analysis for predictors of visual improvement

Variables	OR (CI 95%)	*p*
Age, years	0.983 (0.926–1.043)	0.571
Gender Male Female	Reference 1.750 (0.400-7.664)	0.458
GTV, mm3	0.997 (0.987–1.008)	0.597
Previous treatments Absent Present	Reference 1.833 (0.392–8.566)	0.441
Baseline BCVA, logMAR	0.666 (0.265–1.675)	0.388
Baseline RD Absent Present	Reference 0.121 (0.013–1.120)	0.063
Baseline SRF Absent Present	Reference 0.286 (0.026–3.121)	0.304
Follow-up, months	1.002 (0.972–1.034)	0.882

Abbreviations: *BCVA* best-corrected visual acuity, *CI* confidence interval, *GTV* gross tumor volume, *OR* odds ratio, *RD* retinal detachment, *SRF* subretinal fluid, *UVA* univariate analysis

### Toxicity

**Fig. 4:**
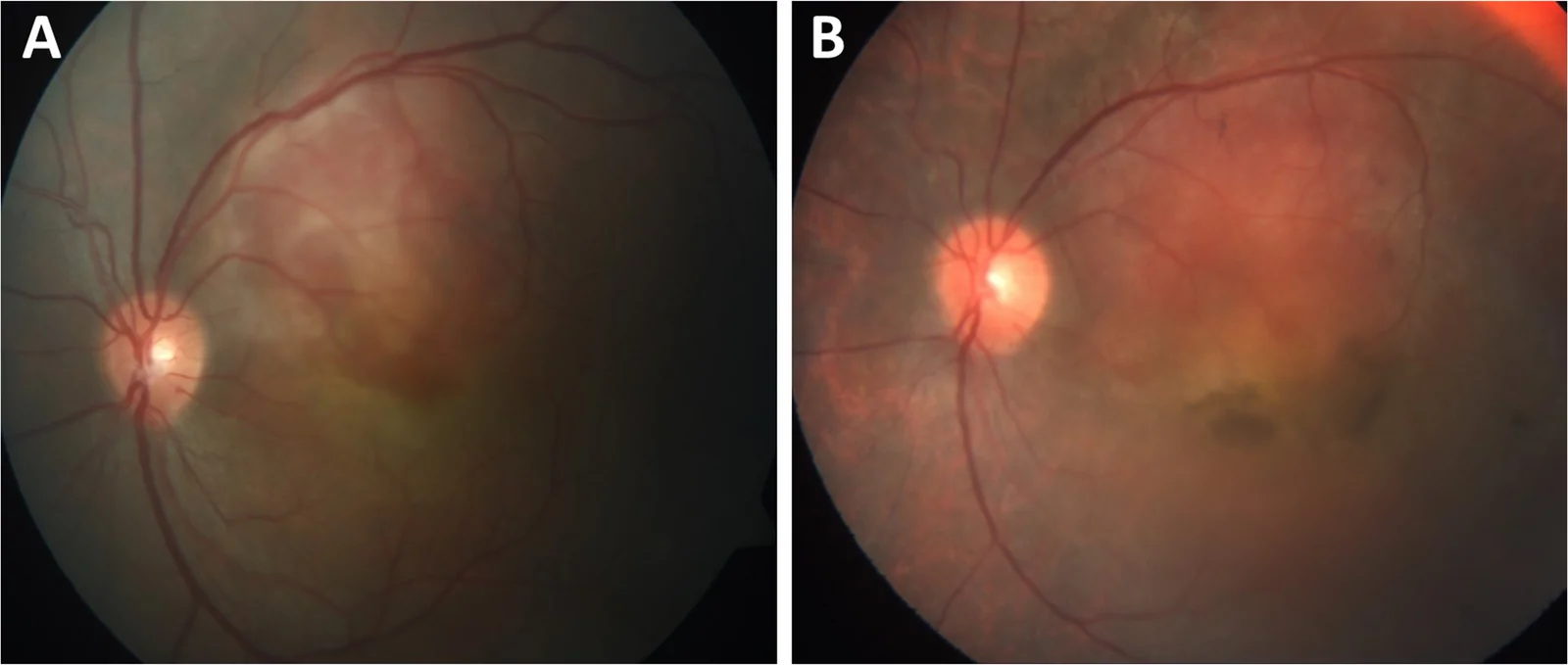
Color fundus photographs of a representative patient before stereotactic radiosurgery (**A**) and at the end of the follow-up period (**B**). Following treatment of the vascular lesion which occupied superior half of the macula, creeping foveal atrophy developed, accompanied by a corresponding decline in visual acuity

No patient experienced any clinically meaningful acute toxicity attributable to SRS. Regarding late toxicities, no cases of radiation-induced cataract were detected during follow-up. A decline in BCVA occurred in two patients (6.7%) after SRS. Both had pre-treatment SRF, and although SRF regressed markedly following treatment, their visual acuity continued to worsen. Radiologically, one patient achieved a complete response and the other a partial response; however, BCVA deterioration persisted in both. Because this decline occurred despite favorable anatomical response, the visual loss was interpreted as late radiation-related retinal injury. In both patients, BCVA worsened from 1.9 to 2.3 logMAR, corresponding to a loss of approximately four lines, and was therefore classified as grade 3 visual acuity decrease according to CTCAE v5.0. Color fundus photography from one of these patients illustrates the development of foveal atrophy during follow-up (Fig. 4). No additional late ocular toxicities were identified in the cohort.

## Discussion

This study demonstrates that SRS provides favorable anatomical and visual outcomes for patients with symptomatic cCH. Although most patients presented with significantly impaired baseline vision, a statistically significant improvement in BCVA was observed following SRS, with the median BCVA improving from 1.9 to 1.3 logMAR. Nearly half of the cohort experienced a clinically meaningful visual gain, while visual stability was achieved in a substantial proportion of the remaining patients. These findings underscore the potential of SRS as an effective treatment option in appropriately selected cases.

Treatment selection for cCH is primarily guided by lesion location, tumor size, and prior treatment history [23]. PDT is generally considered as the first-line approach for most symptomatic patients, owing to its favorable safety profile and efficacy in resolving SRF. TTT may be preferred in small to medium-sized tumors that are not subfoveal, although its thermal nature poses a risk to central vision when used near the macula. Laser photocoagulation, once commonly used, is now largely obsolete due to its limited efficacy and high recurrence rates, and is only occasionally considered for small, extrafoveal lesions. RT, including plaque brachytherapy or EBRT, is typically reserved for refractory cases or large, centrally located tumors where other modalities are either ineffective or carry a higher risk of visual morbidity. The earliest clinical evidence supporting the therapeutic role of RT in cCH was derived from studies utilizing plaque brachytherapy [7, 9–11]. However, its main disadvantages include the requirement for two separate surgical procedures, namely plaque placement and subsequent removal, and the risk of radiation-induced damage to adjacent ocular structures due to limited dose conformality. These limitations have prompted increasing interest in non-invasive, image-guided EBRT techniques such as SRS, which allows for highly conformal dose delivery without the need for surgical intervention in modern clinical practice. Compared to other RT modalities, SRS provides superior spatial precision and real-time tracking, thereby minimizing radiation exposure to surrounding ocular structures. This is particularly advantageous in the treatment of subfoveal or posterior pole lesions, where maximal tissue preservation is critical to maintaining visual function. Proton beam therapy has also been utilized in selected cases of cCH [24]. Its physical dose distribution, characterized by the Bragg peak, allows for sharp distal dose fall-off and theoretically improved sparing of adjacent ocular structures. However, clinical experience with proton therapy in cCH is limited to small case series, and there is currently no evidence demonstrating superior outcomes over photon-based RT techniques [25]. As such, proton therapy remains an alternative option in centers where it is available, particularly for carefully selected cases requiring maximal normal tissue preservation.

To date, most published SRS series for cCH have relied on earlier GammaKnife platforms, which require rigid head frame fixation and are thus considered semi-invasive [4, 12–14]. In contrast, a case report by Agarwal et al. [15] described the use of single-fraction 12 Gy CyberKnife-based SRS in a patient with cCH, demonstrating favorable anatomical and visual outcomes and highlighting the potential of CyberKnife as a fully non-invasive and effective treatment alternative in selected cases. To the best of our knowledge, this is the largest series to date investigating non-invasive SRS in patients with cCH. Based on our findings, SRS appears to be a safe and effective treatment modality, achieving meaningful anatomical and functional improvements in a substantial proportion of patients. Notably, visual improvement was observed in nearly half of the cohort, and high rates of SRF resolution were achieved, underscoring the potential of this non-invasive approach in appropriately selected cases. However, despite these favorable outcomes, we do not propose SRS as the universal first-line treatment for all cCH cases. PDT remains the preferred initial modality for most symptomatic patients. In our practice, SRS is primarily considered for large lesions, tumors with notable chronicity, or highly symptomatic cases, as well as for patients who are refractory to or unsuitable for other established treatments. This approach also reflects the potential risks of radiation-related toxicity to ocular structures, including radiation retinopathy, optic neuropathy, cataract formation, and treatment-related decline in visual acuity [26].

In addition to evaluating the therapeutic efficacy of SRS, our study aimed to identify clinical factors associated with post-treatment visual improvement. Among the variables analyzed, the presence of baseline RD showed a non-significant trend toward reduced likelihood of visual gain. Although this association did not reach statistical significance, it may still hold clinical relevance. RD is often indicative of more advanced disease, typically accompanied by prolonged SRF, accumulation and structural disruption of the outer retina, particularly in the macular region. Such alterations may compromise photoreceptor integrity and limit the potential for functional recovery, even after anatomical reattachment is achieved [27, 28]. Additionally, patients with RD were more likely to have received prior ocular treatments, which may have contributed to cumulative retinal damage and reduced visual potential. These findings suggest that baseline RD may serve as a potential prognostic indicator, and that earlier intervention with SRS, prior to the onset of irreversible retinal injury, could be advantageous in selected patients. However, existing literature presents conflicting evidence. For instance, Kim et al. [12], reported that all seven patients in their study with baseline RD who underwent 10 Gy GammaKnife-based SRS achieved complete anatomical resolution and significant improvement in visual acuity within six months of treatment. Several factors may account for these contrasting outcomes, including differences in the duration and extent of RD, macular involvement, patient demographics, and prior treatment history. Altogether, these observations underscore the complexity of visual prognostication in cCH and highlight the need for further prospective studies to better define the impact of baseline RD and treatment modality on functional outcomes.

Although our study offers meaningful insights into the efficacy of SRS for cCH and the potential clinical predictors of visual outcomes, several limitations should be acknowledged. First, the absence of a control group precludes direct comparisons between SRS and other treatment modalities such as PDT or plaque brachytherapy. Second, while BCVA was consistently assessed using logMAR, more comprehensive evaluations of visual function, including contrast sensitivity and visual field testing, were not available, potentially limiting the functional interpretation of outcomes. Third, follow-up imaging protocols were not standardized, and MRI data were incomplete in some cases, which restricted the ability to perform volumetric analyses of tumor response. Additionally, the relatively small number of patients with baseline RD may have limited the statistical power to detect subtle associations. Finally, the potential confounding effects of prior ocular interventions could not be fully accounted for, which may have influenced both anatomical and visual outcomes following SRS.

In conclusion, SRS appears to be a safe and effective therapeutic option for patients with symptomatic cCH, providing meaningful improvement or stabilization in both anatomical and visual outcomes. While a considerable proportion of patients experienced anatomical improvement, functional recovery was not observed in all cases. The presence of baseline RD showed a trend toward a lower likelihood of visual improvement, suggesting that disease severity at presentation may influence functional outcomes. These findings highlight the importance of timely diagnosis and early treatment before permanent retinal damage occurs. Further prospective studies are needed to confirm these results and to refine patient selection criteria in order to optimize visual outcomes following SRS.

## Data Availability

The datasets generated and analyzed during the current study are not publicly available but are available from the corresponding author (GY) on reasonable request.
